# Pharmaceutical and pharmacokinetic evaluation of novel rectal mucoadhesive hydrogels containing tolmetin sodium

**DOI:** 10.1007/s40005-017-0365-1

**Published:** 2017-11-14

**Authors:** Afaf A. Ramadan, Asmaa M. Elbakry, Asmaa H. Esmaeil, Sahar A. Khaleel

**Affiliations:** 1 Department of Pharmaceutics and Industrial Pharmacy, Faculty of Pharmacy, Al-Azhar University, Nasr City, Cairo, Egypt; 2 Department of Pharmacology & Toxicology, Faculty of Pharmacy, Al-Azhar University, Nasr City, Cairo, Egypt

**Keywords:** Tolmetin, Rectal mucoadhesive hydrogel, Histopathological study, In vitro–in vivo correlation

## Abstract

The objective of the present study was to develop rectal mucoadhesive hydrogels loaded with Tolmetin Sodium, a non-steroidal anti-inflammatory drug, for prolonged duration of action and increased bioavailability. Fourteen formulae were prepared with different types and concentrations of polymers as hydroxypropylmethyl cellulose, hydroxylethyl cellulose, carboxymethyl cellulose and sodium alginate. Each formulation contain Tolmetin Sodium equivalent to 5% w/w active drug. The effect of the employed gel bases on pH, gel strength, mucoadhesion, viscosity and the in vitro release profile of drug was examined. In addition, hydrogel formulations were subjected to rheological and stability studies. The physicochemical characterization revealed that all hydrogels had a suitable pH (6.64–7.75) and gel strength (15.5–65.29 s) for rectal application. The in-vitro drug release from the formulations showed a controlled drug release pattern, reaching 72–92.6% after 8 h. The kinetic analysis of the release data revealed that the drug release from all tested hydrogel bases obeyed the diffusion mechanism. The degradation of Tolmetin Sodium from its rectal hydrogel formulations was found to be a zero-order reaction. All formulations except sodium alginate hydrogel were quite stable. Considering the in-vitro release, rheological properties and shelf life, (CMC; 2%w/w) hydrogel formula was the best among the studied formulations. Therefore, further histopathological and bioavailability studies were carried out to detect different pharmacokinetic parameters of the established formulations compared with commercially available capsules. Formula containing 2% CMC showed relative bioavailability 357.93%. Finally, good correlation was observed between in-vitro and in-vivo profile.

## Introduction

Rectal drug delivery is an efficient alternate to oral and parenteral route of administration in partial avoidance of first pass metabolism and protein peptide drug delivery. This route allows both local and systemic therapy of drugs. Controlled absorption enhancement of drugs can be achieved by the rectal route because of the constant conditions in the rectal environment.

Rectal route offers potential advantages for drug delivery include: rapid absorption of many low molecular weight drugs, reduced side effects such as gastrointestinal irritation, avoidance of disagreeable taste, avoidance of first pass metabolism, potential for absorption into the lymphatic system and possibility of rate controlled drug delivery (Matsuda and Arima 1999; Tukker 2009).

Dosage forms designed for rectal administration should be non-irritant, not cause any damage on mucosal membranes, easy to administer without any pain during insertion, remain at the administration site to avoid the first-pass effect in the liver and the gastrointestinal tract (Kim et al. 1998) and act as mucoadhesive to rectal tissues without leakage after the dose. These requirements could be met by using hydrogels rather than rectal solutions that tend to leak out of the rectum, leading to inaccurate dosing and treatment failure (Amin et al. 2009; Chauhan et al. 2012).

Conventional solid suppository has the disadvantage of providing an alien feeling, discomfort and lowering patient compliance especially when treating a chronic disease. Furthermore, a suppository reaching the end of the colon might lose part of its drug at the colonic level or expose the drug to undergo the first-pass effect (Park et al. 2003). In order to solve the problems of conventional suppository, an attempt was made to develop a hydrogel rectal dosage form has suitable gel strength not to be leaked out from the anus after administration and has a suitable bioadhesive force so as not to reach the end of the colon. Hence, the ideal rectal dosage form should be easy to administer without any pain during insertion and remain at the administration site to avoid the first-pass effect in the liver and the gastrointestinal tract (Kim et al. 1998).

Hydrogels composed of three-dimensional network of hydrophilic polymer chains that could be cross-linked by chemical or physical bonding. Hydrogels are capable of swelling when placed in aqueous media, i.e., they retain a significant amount of water but remain water-insoluble. When a drug substance is loaded into such a hydrogel, the diffusion rate of the drug depends on the physical structure of the polymer network and its chemical nature (Dimitrov et al. 2003).

Hydrogels may offer several advantages (Amin et al. 2009; Chauhan et al. 2012): sustained and prolonged action in comparison to conventional drug delivery systems, decreased dose of administration, decreased side-effects, drug targeting to specific site like colon, protection of mucosa from irritating drugs, drug loss is prevented by extensive first pass metabolism, lower daily cost to patient due to fewer dosage units are required by the patient in therapy and drug adapts to suit circadian rhythms of body functions or diseases.

Tolmetin Sodium is a pyrrole, acetic acid derivative, non-steroidal anti-inflammatory drug commonly used for the treatment of rheumatoid arthritis, osteoarthritis, ankylosing spondylitis and periarticular disorders. It inhibits cyclooxygenase activity with a reduction in the tissue production of prostaglandins. Its usefulness is limited due to its short plasma half-life of 30–60 min following oral dosing, which necessitates frequent administration of the drug in order to maintain the desired steady state levels (Katzung 2004). Tolmetin Sodium considers a safe alternative for multiple NSAID-concomitant acetaminophens (Ozlem et al. 2013).

The present study aimed to prepare and evaluate rectal application mucoadhesive hydrogels as delivery system for Tolmetin Sodium to achieve prolonged duration of action and increased bioavailability. The rheological behaviors, mucoadhesiveness, in vitro release of drug were studied. Additionally, histopathological and pharmacokinetic studies were evaluated. Finally, correlation between in-vitro and in-vivo data (IVIVC) was done.

## Materials and methods

### Materials

Tolmetin Sodium was kindly supplied by Sigma Pharma (Egypt). Hydroxypropylmethyl cellulose (HPMC K4M) was purchased from Dow Chemical Co. (USA). Carboxymethyl cellulose (CMC) was kindly supplied by Eipico Pharma (Egypt). Hydroxyethyl cellulose (HEC) was kindly supplied by Delta Pharma (Egypt). Sodium alginate was purchased from El-Nasr Pharmaceutical Co. (Egypt). Cellulose membrane (molecular weight cut-off 10,000) was purchased from Sigma Chemical Co. (USP). Male albino rats weighing 200–220 g were obtained from animal house (Al-Azhar University, Egypt).

### Preparation of Tolmetin rectal hydrogels

The composition of Tolmtin Sodium rectal hydrogel formulae are shown in Table 1.

**Table 1:** Composition and physical characters of prepared Tolmetin sodium hydrogels (5% w/w)

Formula	Polymer	Polymer conc. (% w/w)	pH ± SD	Drug content ± SD	Gel strength ± SD (second)	Viscosity(cp) ± SD	Mucoadhesive Force (dyne/cm^2^) ± SD
F1	HPMC	3	6.90 ± 0.11	101.75 ± 0.34	27.60 ± 2.04	13,917 ± 221	2146 ± 166.7
F2		5	6.64 ± 0.09	100.22 ± 0.50	35.76 ± 0.73	55,784 ± 519	3012 ± 132.4
F3		7	6.93 ± 0.13	99.98 ± 1.35	40.50 ± 1.76	99,537 ± 129	3621 ± 301.3
F4		10	7.31 ± 0.11	102.30 ± 0.36	53.67 ± 1.43	247,394 ± 1105	4357 ± 136.4
F5	HEC	2	6.82 ± 0.10	102.78 ± 0.23	38.50 ± 0.83	64,372 ± 214	2746 ± 130.4
F6		3	6.84 ± 0.09	101.39 ± 0.84	46.60 ± 0.55	335,037 ± 403	3466 ± 150.5
F7		4	7.25 ± 0.80	99.87 ± 0.85	62.58 ± 1.62	556,201 ± 826	5343 ± 105.6
F8	CMC	2	6.86 ± 0.05	100.75 ± 0.46	36 ± 2.75	49,320 ± 641	3027 ± 187.2
F9		3	6.85 ± 0.07	100.46 ± 1.54	49.75 ± 1.06	109,546 ± 894	3664 ± 212.8
F10		4	7.00 ± 0.11	99.90 ± 0.58	65.29 ± 3.09	*	6758 ± 521.9
F11	Alginate	5	7.39 ± 0.09	99.45 ± 1.89	15.5 ± 2.26	27,492 ± 531	1600 ± 241.7
F12		7	7.52 ± 0.06	98.29 ± 0.97	24 ± 1.55	66,212 ± 364	2458 ± 246.6
F13		10	7.62 ± 0.10	100.45 ± 2.12	33 ± 0.98	142,468 ± 215	4107 ± 231.5
F14		15	7.75 ± 0.06	100.60 ± 0.67	43.33 ± 1.50	181,076 ± 643	6500 ± 379.8

All values are Mean ± SD, (n = 3)

*Not detected

### Preparation of HPMC, HEC and CMC hydrogel bases

Tolmetin was dissolved in the calculated amount of hot distilled water. The calculated amounts of each HPMC (3, 5, 7, 10% w/v), HEC (2, 3, 4% w/v) and CMC (2, 3, 4% w/v) were separately weighed and added gradually to the drug solution with gentle stirring (120 rpm) using a magnetic stirrer (Dubuque, Iowa, USA). Stirring was continued until no lumps were observed and the content left overnight in refrigerator (4 °C) to complete solubility and gel formation (Auda et al. 2015).

### Preparation of sodium alginate hydrogel base

Tolmetin was dissolved in the calculated amount of distilled water. The calculated amounts of sodium alginate (5, 7, 10, 15% w/v) was sprinkled to drug solution and the method of preparation was completed as with cellulose derivatives (Tasdighi et al. 2012).

## Evalution of hydrogel formulations

### Color and homogeneity

All prepared medicated hydrogels were tested for homogeneity by visual inspection after the hydrogels have been set in the container.

### Determination of pH of prepared hydrogels

The pH of each medicated hydrogel formulations was determined using a pH meter (410A, ORION). Solution containing 1 g of each formula in 30 ml of distilled water was prepared and the pH was measured. The pH measurements were triplicated (Shivhare et al. 2009).

### Determination of drug content

A specified quantity (1 g) of developed hydrogel formula was dissolved in 100 ml of phosphate buffer (PB pH 6.8). The obtained solution was filtered through a Millipore filter (0.45 µm) and estimated spectrophotometrically at 324 nm using (PB pH 6.8) as blank. The concentration of drug in sample was calculated (Baviskar et al. 2013).

### Measurement of gel strength

**Fig. 1:**
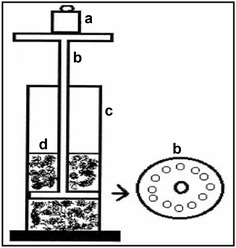
Gel strength measuring device. *a* Weights; *b* device; *c* glass cylinder; *d* Tolmetin hydrogel

The gel strength of Tolmetin Sodium rectal hydrogel formulations was determined by using the gel strength measuring device shown in Fig. 1. A sample of 50 g of prepared hydrogel was placed in a 100 ml graduated cylinder. A standard weight of 35 g was placed onto the hydrogel surface. The strength of gels was determined by measuring the time in seconds taken by the weight to penetrate 5 cm down through the gel. A range of 10–50 s was acceptable for rectal application. A time less than 10 s was considered to cause the leakage out from the rectum, whereas more than 50 s would be too viscous for rectal administration (Choi et al. 1998).

### Measurement of the mucoadhesive force

**Fig. 2:**
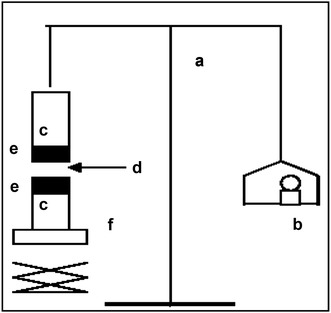
Mucoadhesive force measuring device. *a* Modified balance; *b* weights; *c* glass vial; *d* Tolmetin hydrogel; *e* Tissue; *f* height adjustable pan

The mucoadhesive force of the Tolmetin rectal hydrogels was determined by using the mucoadhesive force measuring device shown in Fig. 2, using tissues cut from the fundus of rabbit rectum. The pieces of tissues were stored frozen in sorenson^’^s phosphate buffer pH 7.4 and thawed to room temperature before use. At the time of testing a section of tissue was secured with mucosal side out onto each glass vial (C) using a rubber band and aluminum cap. The diameter of each exposed mucosal membrane was 2 cm. The vials with the rectal tissue were stored at 37 °C for 10 min. Next, one vial with a section of tissue (E) was connected to the balance (A) and the other vial was placed on a height-adjustable pan (F). Tolmetin hydrogel (D) was added onto the rectal tissue on the other vial. Then, the height of the vial was adjusted so that the hydrogel could be placed between the mucosal tissues of both vials. The weights (B) were increased until the two vials were detached. Bioadhesive force, the detachment stress (dyne/cm^2^), was determined from the minimal weights that detached the two vials.

The following formula was used to determine mucoadhesive force in (dyne/cm^2^):

$${\text{Detachment stress }}\left( {{\text{dyne}}/{\text{c}}{{\text{m}}^{\text{2}}}} \right){\text{ }}={\text{ }}\left( {\text{m}} \right){\text{ }} \times {\text{ }}\left( {{\text{g }}/{\text{A}}} \right)$$

where, m = weight required for detachment of two membrane in grams, g = acceleration due to gravity [980 cm/s^2^], A = area of tissue exposed.

The mucosa was changed for each measurement. Measurements were repeated three times for each of the gel preparations (Jadhav et al. 2009).

### In-vitro release studies

The in-vitro release of Tolmetin Sodium from rectal hydrogels was monitored using franz diffusion cells with cellophane membrane placed between the donor and the receptor compartment. The membrane was soaked in PB pH 6.8 overnight and then washed before use. A known amount (1 g) of each hydrogel formulations containing 50 mg Tolmetin was added to the donor side and a known volume of PB pH 6.8 (500 ml) was added to receptor side. The receptor compartment were maintained at 37 ± 0.5 °C throughout the experiment. Five-milliliter samples were withdrawn at predetermined time over an 8-h period from the receptor compartments and analyzed spectrophotometrically at *λ*_max_ 324 nm. The withdrawn samples were replaced immediately with an equal volume of fresh buffer. The percent cumulative amount of drug released was calculated. All experiments were carried out in triplicate and the average values were calculated ± the standard deviation (Abd El-Rasoul et al. 2012).

### Kinetic analysis of drug release data

To understand the mechanism of drug release of different formulae of hydrogels, The in-vitro release data of drug from the investigated hydrogel formulations were studied using a special computer program (provided kindly by Al-Azhar University, faculty of pharmacy, girls) according to zero-order kinetic model, first-order and Higuchi’s model. Further, the dissolution data were fit to the Koresmeyer-Peppas model, to analyze drug release mechanism from polymeric systems (El-Leithy et al. 2010).

$${{\text{M}}_{\text{t}}}{\text{=Kt for zero-order kinetics}}$$

$${\text{Ln M }}={\text{Ln }}{{\text{M}}_0}-{\text{ Kt for first-order kinetics}}$$

$${{\text{M}}_{\text{t}}}/{{\text{M}}_\infty }={\text{K}}{{\text{t}}^{{\text{1}}/{\text{2}}}}{\text{ for Higuchi}}\text{'}{\text{s model}}$$

$${{\text{M}}_{\text{t}}}/{{\text{M}}_0}={\text{K}}{{\text{t}}^{\text{n}}}{\text{ for Koresmeyer-Peppas model}}$$

where, M_0_ is the initial amount of drug released at zero time, M_t_ is the amount of drug released at time t, M_t_/M_∞_ is the fraction of drug released at time t, K is kinetic constant characteristics for drug/polymer and “n” is release exponent that characterize the drug transport or it is the diffusional exponent indicative of the mechanism of drug release (Kuksal et al. 2006). For Korsmeyer-Peppas equation, If “n” ≤ 0.5, the release mechanism is represented by Fickian Diffusion (Higuchi matrix); if 0.5 < “n” < 1, it suggests anomalous transport (non-Fickian) transport due to both drug diffusion and polymer chain relaxation, for “n” = 1, the zero order release is possible and if “n” > 1, a super Case II transport is operative (Pasa et al. 2012).

### Rheological studies

The viscosity of the medicated hydrogel bases containing 5% Tolmetin Sodium was determined using a programmable viscometer (Brookfield apparatus, Inc DV-II+Pro Viscometer). The measurement of the viscosity was done on each medicated base separately at room temperature (25 ± 1 °C) with 1 min interval. The angular velocity was changed from 0.2 to 2 rpm at controlled ramp speed. The average of three readings was used to determine the rheological parameters; the viscosity (cp), shear rate (1/s) and shear stress (dyne/cm^2^) were determined (Mekkawy et al. 2013). Flow behavior was further analyzed by regression analysis of the log shear stress versus log shear rate and the following equation was applied:

$${\text{Log G }}={\text{ n Log F }}-{\text{ Log }}\upeta$$

where, G is shear rate (s^−1^), F is shear stress (dyne/cm^2^), ɳ is viscosity (cp), and n is the farrow^ʹ^s constant and is considered an index of the deviation from newtonian flow behavior. The more the value of n differs from unity the more non-newtonian is the flow behavior. For pseudoplastic flow, “n” > 1 while for dilatancy, “n” < 1.

### Measurement of thixotropy

Thixotropy is a desirable property in most of pharmaceutical systems. Measurement of thixotropy for each medicated hydrogel was determined using curve expert program in order to calculate the hysteresis loop between upward and downward curve for the relationship between shear rate and share stress (Abdallah et al. 2010).

### Histopathological study

The selected hydrogel formulations [F8 (2% HEC) & F11 (2% CMC)] containing 5% Tolmetin Sodium as pure drug were tested for abnormal irritability on the rat rectal mucosa. Nine male albino rats weighing 200–220 g were randomly divided into three groups (three animals per group). Group I was the control group, group II was for the pure drug rectal solution and Group III was for drug incorporated in a 2% w/w CMC hydrogel (F8). The animals were fasted for 24 h prior to the experiment but allowed free access to water. All institutional and national guidelines for the care and use of laboratory animals were followed. The amount of drug for all tested groups was equivalent to 50 mg/kg and administrated into the rectum of rats, 4 cm above the anus, using a sonde fitted to a plastic syringe. Control animals injected rectally with normal saline. The entrance of the anus was then blocked with a cyanoacrylate adhesive to prevent leakage out of hydrogel or saline from the anus. After 8 h of rectal administration for the different dosage forms, the animals were sacrificed and their rectal segments were isolated, and then immersed in 10% v/v formalin buffer for 24 h. The segments prepared for examination by washing with distilled water then serial dilutions of alcohol (methyl, ethyl and absolute ethyl) for their dehydration. Specimens were cleared in xylene and embedded in paraffin at 56 degree in hot air oven for 24 h. Paraffin bees wax tissue blocks were prepared for sectioning at four microns thickness by slidge microtome. The obtained tissue sections were collected on glass slides, deparaffinized, stained by hematoxylin and eosin stains. A light microscope was used for histopathological abnormalities examination such as inflammatory cell infiltration. The abnormality was quantified on an arbitrary scale from 0 (no effect) to 2 (severe effect) (El-Leithy et al. 2010; Ban and Kim 2013).

### Pharmacokinetic study

Twelve male albino rats weighing 200–220 g, randomly divided into two groups, were used in this study as follows:

- Treatment (I): Using commercial capsule containing 200 mg Tolmetin Sodium namely Rumatol^®^ capsule (Sigma Pharma).
- Treatment (II): using formula (F11) rectal hydrogel containing 5% Tolmetin Sodium and 2% CMC.

Measurement of pharmacokinetic parameters of the treatments conformed to guide lines of Institutional Animal Ethical of Al-Azhar University. The rats in each treatment were fasted from food 24 h prior to dosing. Rats in treatment (I) group were administered a single oral dose of Tolmetin Sodium capsule (Tolmetin equivalent to 50 mg/kg in suspension form). Rats in treatment (II) were administered a single rectal dose of Tolmetin Sodium (F11) hydrogel (Tolmetin equivalent to 50 mg/kg) using a sonde fitted to a plastic syringe. Blood samples were collected from retro-orbital vein of rats using heparinized tubes following time intervals: 0 (pre-dose), 0.5, 1, 2, 4, 8, 12, and 24 h post oral dose of commercial capsule suspension and rectal dose of prepared rectal, Tolmetin hydrogel, then immediately centrifuged at 3000 rpm for 20 min. The plasma was separated in screw capped tubes via micropipette and frozen at -20 °C until assayed. A conventional HPLC instrument was used to detect Tolmetin in rat plasma, as rapid, sensitive and selective method (Loya and Saraf 2010).

### In vitro–in vivo correlation

In-vitro and in-vivo correlation was carried out to compare the release of drug. It is governed by the factors related to both in-vitro and in-vivo characteristics of the drug. The cumulative percentage of drug releases both in-vitro and in-vivo was plotted (Prasanna and Sankari 2012).

## Results and discussion

### Evaluation of the hydrogel loaded with Tolmetin Sodium

The formulated hydrogels exhibit transparent, yellow color with a smooth and homogenous appearance.

#### pH

The formulated hydrogels had pH values in range of 6.64–7.75. This pH range is close to the pH of rectum (6.8). This indicates the suitability of the hydrogels for rectal application with minimal risk of tissue irritation.

#### Drug content

The determined drug content values were ranged from 98.29 to 102.78 ± 5% SD. The difference in drug content from different formulations may be due to their different pH and electrolyte in their formulations.

#### Gel strength

In the development of rectal hydrogel, the gel strength is important in finding the condition that allow easy and no retaining of hydrogel with no leakage from the anus (Kim et al. 1998). The measured gel strengths of the prepared formulae were shown in Table 1. All mucoadhesive polymers abruptly increased the gel strength as the concentration increased. This variation in the gel strength of Tolmetin Sodium hydrogels may attribute to variation in nature and composition of mucoadhesive polymers used in gel preparation.

#### Muco-adhesion force

Mucoadhesive force is force required to detach the formulation from mucosal surface. Mucoadhesive force is known to be dependent on the nature and the concentration of mucoadhesive polymers. The stronger the mucoadhesive force is, the more it can prevent the hydrogels from reaching the end of the colon. If the mucoadhesive force is too excessive, the gel can damage the rectal mucous membrane (Abu El-Enin and El-Feky 2013). The mucoadhesive strengths of Tolmetin Sodium hydrogel formulations were shown in Table 1. It was evident that, formulations containing the same polymer with different concentrations, as the concentration of the polymer increased the mucoadhesive force increased. This phenomenon can be attributed to the fact that, at lower concentration of the polymer chains, there is an inadequate and unstable interaction between the polymer and the mucosal layer resulting in lower mucoadhesive properties (Roy et al. 2009). Tolmetin Sodium hydrogel formulations containing CMC showed higher mucoadhesive forces as compared to formulations containing HPMC and HEC. This finding can be explained by presence of charged functional groups (negatively charged carboxyl groups) in polymer chains of CMC, may render it as polyelectrolytes and this has a marked effect on the strength of mucoadhesion due to the formation of strong hydrogen bonds between the polymer functional groups and the mucosal layer as compared to neutral polymers such as HPMC and HEC. In general, anionic polyelectrolytes have been found to form stronger mucoadhesive bond when compared to the neutral ones (Tasdighi et al. 2012).

#### In-vitro release of Tolmetin Sodium hydrogels

**Fig. 3:**
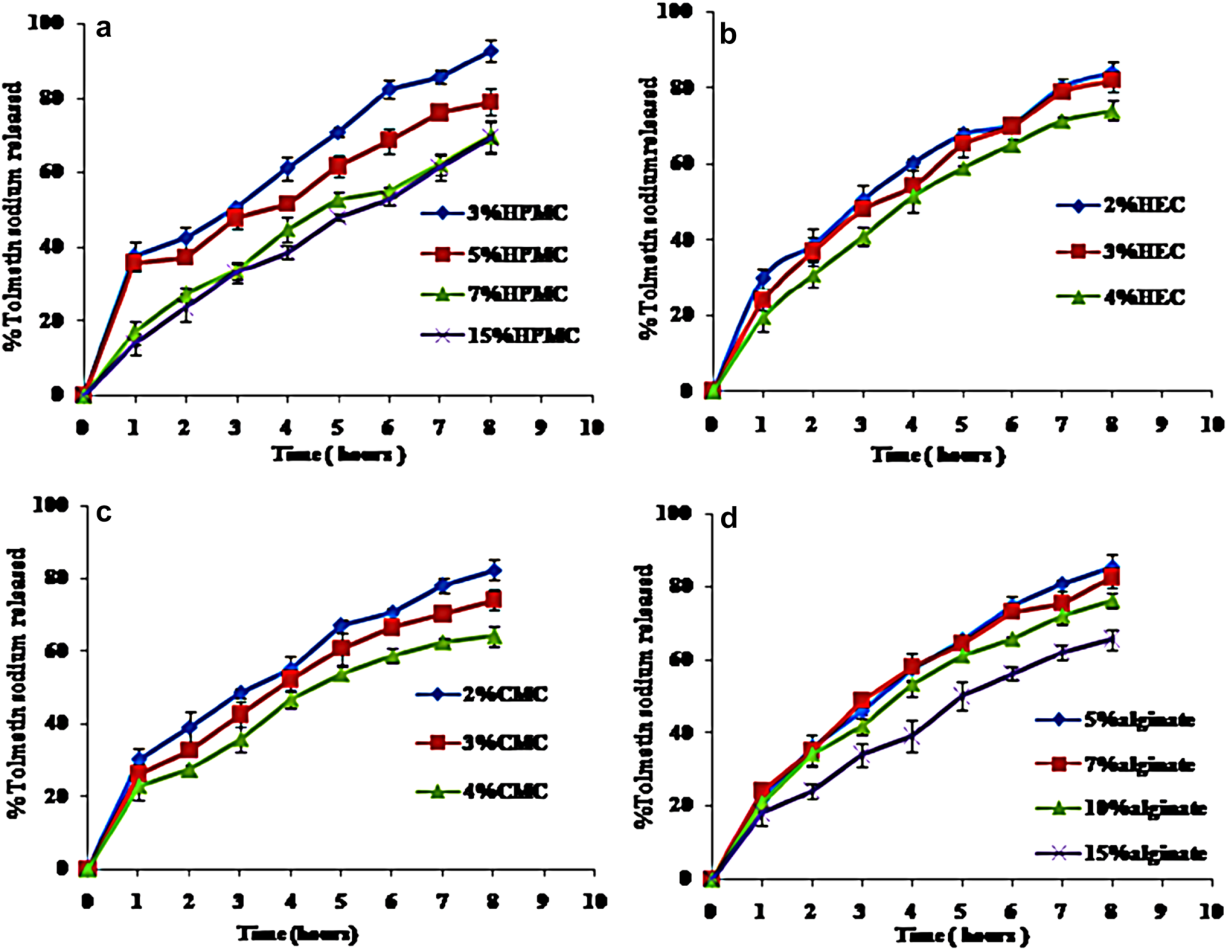
In-vitro release of Tolmetin Sodium from different hydrogel bases, **a** HPMC, **b** HEC, **c** CMC and **d** sodium alginate, through cellulose membrane into PB pH 6.8

The in-vitro release profile of Tolmetin Sodium from the prepared hydrogels was studied using PB pH 6.8 as receptor medium for 8 h, to detect the effect of type and concentration of the gelling agent in different concentrations on the drug release. The effect of mucoadhesive polymer type and concentration on the release of Tolmetin Sodium from the prepared hydrogels are illustrated in Fig. 3. It is clear that the release of Tolmetin Sodium varies from 92.64 to 64% after 8 h depending on type and concentration of gel base. It was found that drug release was retarded with higher polymer concentrations, such retardation in the release rate can be attributed to increasing the concentration of swellable polymers which increased the product viscosity and thereby decreased the rate of penetration of dissolution medium. It was also possible that at higher polymer concentration, the active substance was trapped by the polymer molecules. This increased the resistance to diffusion more than expected. Additionally the density of chain structures which has been observed in gels microstructure increased at higher polymer concentrations and this limits the active substance movement area (Abdallah et al. 2010).

#### In-vitro release kinetic parameters of Tolmetin Sodium hydrogels

In order to develop an ideal kinetic model to interpret the diffusion data in terms of meaningful parameters, various kinetic models including zero-order, first-order, and Higuchi models were applied to obtain the best fit for the results. As shown in Table 2, it was found that in vitro release of Tolmetin Sodium hydrogels followed non-Fickian diffusion transport; this indicated that the drug release mechanism may involve a combination of both diffusion and chain relaxation mechanism. Therefore, the release of drug from the formulated gels is controlled by swelling of the polymer, followed by drug diffusion through the polymer and slow erosion of polymer (Dias et al. 2009).

**Table 2:** Kinetic parameters for the in-vitro release of Tolmetin Sodium from different rectal hydrogel formulations

Formula	Kinetic order or model	Intercept (a)	Slope (b)	Correlation coefficient (r^2^)	Rate constant	t_1/2_ (min)
F1	Diffusion	− 11.89	36.8284	0.9960	36.8284	1.84
F2	Diffusion	− 16.59	37.1965	0.9952	37.1965	1.86
F3	Diffusion	− 13.4085	28.7874	0.9955	28.7874	3.0167
F4	Diffusion	− 18.2087	29.8460	0.9978	29.8460	2.8065
F5	Diffusion	− 1.9303	30.5330	0.9963	30.5330	2.6816
F6	Diffusion	− 8.5919	32.3963	0.9980	32.3963	2.3820
F7	Diffusion	− 12.0429	31.2065	0.9978	31.2065	2.5671
F8	Diffusion	− 1.7439	29.6732	0.9960	29.6732	2.8393
F9	Diffusion	− 4.5108	28.2060	0.9942	28.2060	3.1423
F10	Diffusion	− 4.9226	25.2104	0.9874	25.2104	3.9335
F11	Diffusion	− 14.8179	35.9084	0.9989	35.9084	1.9388
F12	Diffusion	− 8.2646	32.3351	0.9966	32.3351	2.3910
F13	Diffusion	− 9.6655	30.8245	0.9980	30.8245	2.6311
F14	Diffusion	− 13.0367	27.7746	0.9935	27.7746	3.2407

#### Rheological properties of Tolmetin Sodium hydrogels

**Fig. 4:**
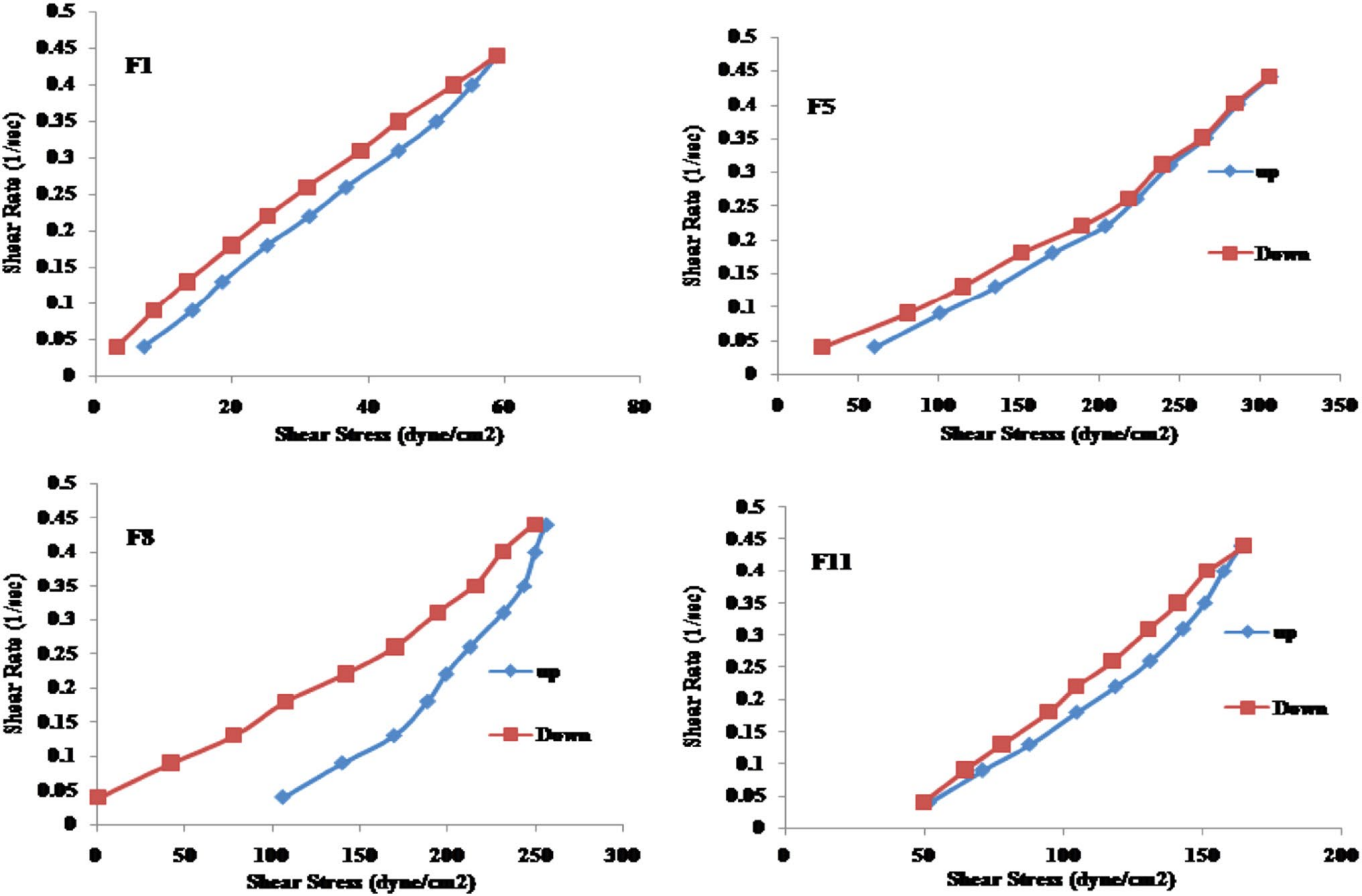
Rheological behavior of Tolmetin Sodium hydrogel containing different polymers, **F1** (3%HPMC), **F5** (2%HEC), **F8** (2%CMC) and **F11** (5% sodium alginate)

The viscosity of rectal hydrogels had an influence on the rate of drug release and distribution in the distal portion of the large intestine. In addition, the relative viscosity could provide some insight about the expected mucoadhesive strength of the gel and its retention time. Moreover, the evaluation of rheological properties of the formulation as a dosage form would be important for predicting their behavior in-vivo (Chang et al. 2002). The rheological properties of hydrogel formulations in the tested experimental conditions were shown in Table 3 and illustrated graphically in Fig. 4. The prepared formulations exhibited pseudoplastic rheology, as evidenced by shear thinning and an increase in the shear stress with increasing the angular velocity which confirmed the hydrogels pseudoplastic properties. It was noted that, the viscosity of the medicated hydrogels increased upon increasing the concentration of mucoadhesive polymer, and some of these medicated hydrogels exhibited high viscosity to the extent that affect negatively on the spindle of the Brookfield’s Apparatus. These formulations were F4 (containing 10% HPMC K4M), F7 (containing 4% HEC), F10 (containing 4% CMC), and F13 and F14 (containing 10 and 15% sodium alginate, respectively). Thus, these formulations were excluded upon comparing the rheograms for the medicated hydrogel under test. Concerning the rheograms for the rest of the medicated hydrogel formulations, they all exhibited pseudoplastic flow without thixotropy except four formulations which exhibited thixotropy. These formulations are F1 (containing 3% HPMC K4M), F5 (containing 2% HEC), F8 (containing 2% CMC) and F11 (containing 5% sodium alginate). To study the flow behavior of the different medicated hydrogels, (four formulae with thixotropy), farrow’s equation was applied. The calculated farrow’s values were greater than one, F1 (3.19), F5 (1.9), F11 (1.77), F8 (1.6) which confirmed the hydrogels pseudoplastic properties. Upon comparing the different formulae, it was observed that one prepared with 2% CMC (F8) had highest shear thinning effect. In addition, the area of its hysteresis loop was the largest indicating a better thixotropic behavior. Thus, this formula was selected for rectal administration and for further histopathological evaluation.

**Table 3:** Viscosity and thixotropic behavior of Tolmetin Sodium hydrogels

Formula	Viscosity (cp)	Thixotropic behavior (cm^2^)	Farrow’s constant
F1	13,917	3.82	3.19
F5	64,372	2.11	1.9
F8	49,320	5.57	1.6
F11	27,492	3.11	1.77

#### Histopathological study

The histopathological examination was performed to study the effect of Tolmetin Sodium on the rat rectal mucosa when applied as pure drug form or incorporated in CMC (2% w/w) hydrogels. The rectal tissue at 8 h after rectal hydrogel administration to male albino rats was observed and compared with untreated normal tissue. Visual inspection of the rectal mucosa of the rat groups after 8 h of rectal administration of the hydrogel formulae revealed attachment of a fraction of the inserted dose to the mucosa. This adhesion property of the hydrogels to the site of application prevented the hydrogels from reaching the upper part of the rectum, thus, considered an advantage in avoiding the first pass effect.

**Fig. 5:**
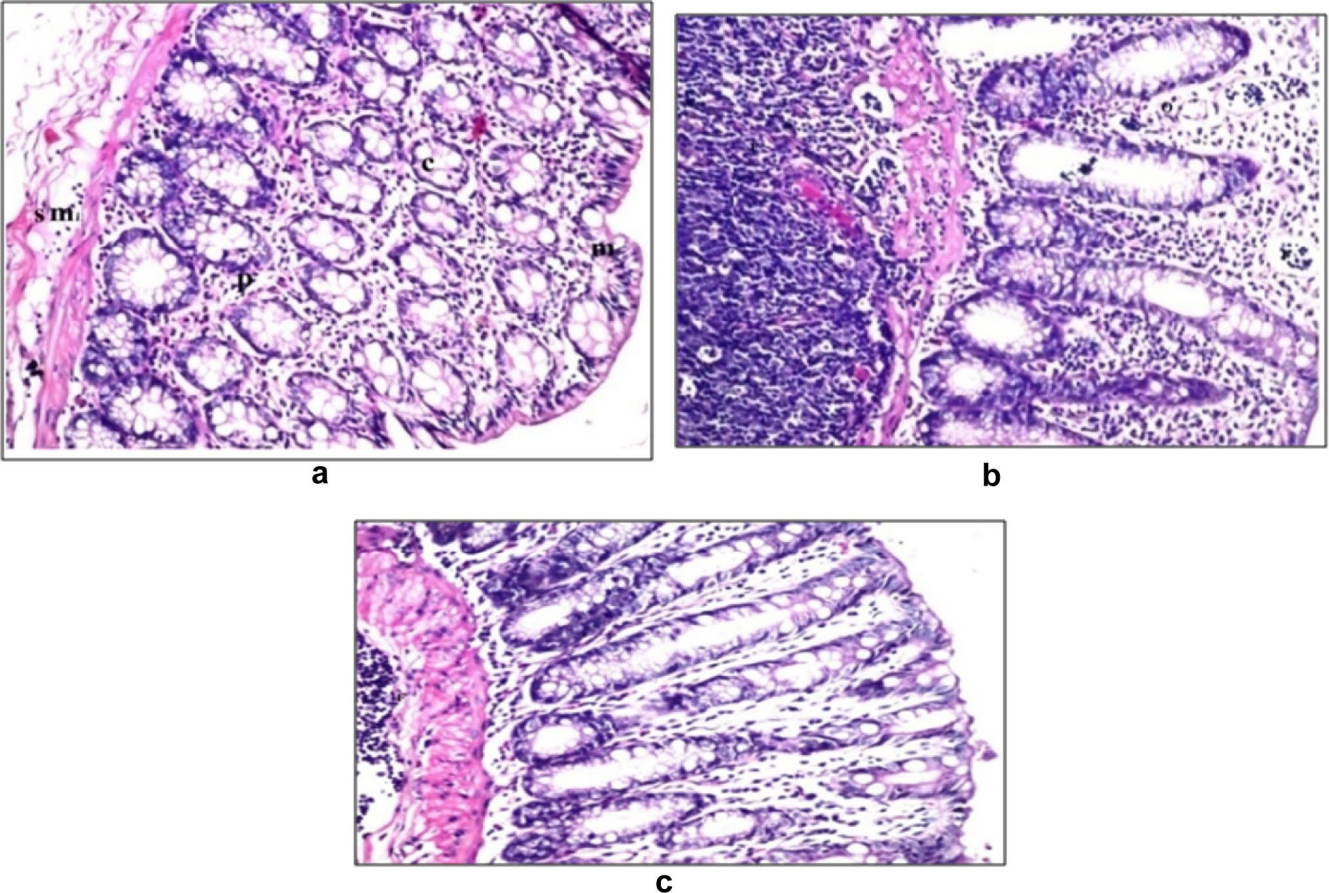
Histopathological examination of **a** untreated rat rectal mucosa in comparison to 8 h after rectal administration of **b** Tolmetin solution, **c** Tolmetin Sodium incorporated in 2% CMC hydrogel (F8). *F stands for inflammatory cells and *O* stands for edema

The results of the histopathological evaluation were shown in Fig. 5.

- Group I: The negative control group (Group I), showed a normal Histological rectal mucosa with a series of small longitudinal folds of large and broad mucosal crypts. The mucosal layer was also lined with simple columnar epithelial cells containing few amounts of goblet cells, Fig. (5a).
- Group II: This group includes rats that were administered rectally Tolmetin Sodium as rectal solution. The lamina propria of the mucosa showed massive number of inflammatory cells infiltration associated with lymphoid hyperplasia in the follicles at the submucosa. Edema with focal inflammatory cells aggregation as well as infiltration was detected also in the submucosa, Fig. (5b).
- Group III: This group includes rats that were administered rectally F8 that contain Tolmetin Sodium incorporated in 2% CMC hydrogel. Few inflammatory cells infiltration was detected in the lamina propria of the mucosa with absence of edema while the submucosa had few lymphoid hyperplasia, Fig. (5c).

These results may be attributed to the highest inflammatory response caused by Tolmetin Sodium crystalline structure, while Incorporation of the drug in hydrogel form reduce this inflammatory response due to soft rubbery nature of hydrogel which minimizes mechanical and frictional irritation to the surrounding tissues.

#### Pharmacokinetic study

**Fig. 6:**
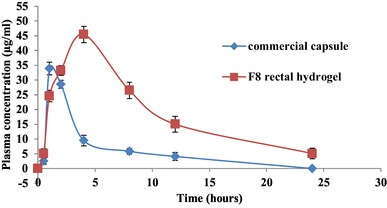
Tolmetin Sodium mean plasma concentration-time curve after oral administration of Rumatol capsule and rectal administration of F8 hydrogel to six rats

The rectal absorption of Tolmetin was evaluated when Tolmetin Sodium hydrogel was administered via the rectum. Oral and rectal administrations are shown in Fig. 6. The pharmacokinetic parameters are shown in Table 4. Tolmetin concentration increased rapidly after oral administration and yielded the maximum plasma concentration C_max_ within 1 h. On the other hand, rectal administration of Tolmetin Sodium hydrogel showed a slow absorption profile for Tolmetin and C_max_ was reached after 4 h. The t_1/2_ was greater in Tolmetin Sodium hydrogel group. Therefore, prolonged release of Tolmetin may be due to the mucoadhesive gel matrix of CMC. Tolmetin Sodium hydrogel provided a significantly higher AUC_0−24_ for Tolmetin as compared to the oral control group. The percentage relative bioavailability for hydrogel formula was found to be 357.93%. Improved bioavailability of Tolmetin could be attributed mainly to the avoidance of hepatic first-pass effect, which was a consequence of drug retention in the lower rectum, by the aid of the mucoadhesive polymer. Our results suggest that Tolmetin Sodium rectal hydrogel would be useful for Tolmetin delivery, allowing easy self-administration by patients and avoiding the first pass effects.

**Table 4:** Pharmacokinetic parameters of Tolmetin Sodium estimated after oral and rectal administration of commercial capsule suspension (treatment I) and F8 hydrogel (treatment II) to six rats

Parameter	Commercial capsule	Rectal hydrogel (F8)
Dose (mg/kg)	50	50
C_max_ (µg/ml)	33.877 ± 2.16	45.425 ± 2.745
t_max_ (h)	1	4
t_1/2el_ (h)	5.137 ± 0.027	6.910 ± 0.003
AUC_0−24_ (µg h/ml)	129.489 ± 13.55	463.481 ± 48.207
AUC_0−∞_ (µg.h/ml)	129.489 ± 13.55	514.033 ± 48.207
MRT (h)	3.923 ± 0.24	9.512 ± 0.233
Relative bioavailability	–	357.93%

All values are Mean ± SD

#### In vitro–in vivo correlation

**Fig. 7:**
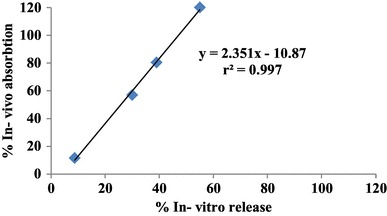
Level A in vitro–in vivo correlation for Tolmetin Sodium rectal hydrogel formula (F8)

Good correlation was observed between in-vitro and in-vivo profile, revealed the ability of the formulation to reproduce the in-vitro release pattern through the biological membrane. Linear regression analysis was applied to the in vitro-in vivo correlation plots and coefficient of correlation (r^2^), slope and intercept values were calculated and are presented in Fig. 7. The correlation coefficient (r^2^) for IVIV correlation level A was 0.997. Hence, Tolmetin Sodium mucoadhesive rectal hydrogel could be promising one as they increase bioavailability, minimize the dose, reduces the side effects, and improve patient compliance (Ramesh et al. 2015).

## Conclusion

The HPMC, HEC, CMC and alginate hydrogels containing Tolmetin Sodium have been prepared and evaluated. The formula of 2% w/w CMC loaded with Tolmetin Sodium (F8) showed adequate rheological characteristics and mucoadhesive properties. The suggested formula allowed the adhesion of the hydrogel loaded with the drug to the rectal mucosa for subsequent controlled release behavior with no burst effect. Histopathological evaluation of the selected formula revealed the effectiveness of incorporation of anti-inflammatory drugs into the hydrogel delivery system in reducing the irritation to the mucosal tissues. The relative bioavailability of our promising formula was 357.93% compared to commercial capsule; Thus Tolmetin Sodium might be a right and suitable candidate for rectal controlled drug delivery via mucoadhesive rectal hydrogels.

## References

[CR1] Abd El-Rasoul S, Auda SH, Nafady AM (2012). In vitro and in-vivo studies of tolmetin release from natural gel base extracted from okra seed (*Abelmoschus Esculentus*) that cultivated in Egypt. Glob J Med Res.

[CR2] Abdallah FI, Dawaba HM, Mansour AM, Samy AM (2010). Preparation, characterization and stability studies of piroxicam loaded microemulsions in topical formulations. Drug Discov Ther.

[CR3] Abu El-Enin ASM, El-Feky GS (2013). Formulation and evaluation of in-situ gelling tenoxicam liquid suppositories. J Life Med.

[CR4] Amin S, Rajabnezhad S, Kohli K (2009). Hydrogels as potential drug delivery systems. Sci Res Essay.

[CR5] Auda SH, Abd El-Rasoul S, Ahmed MM, Osman SK, Mahmoud El-Badry M (2015). In-vitro release and in-vivo performance of tolmetin from different topical gel formulations. J Pharm Investig.

[CR6] Ban E, Kim CK (2013). Design and evaluation of ondansetron liquid suppository for the treatment of emesis. Arch Pharm Res.

[CR7] Baviskar DT, Biranwar YA, Bare KR, Parik VB, Sapate MK, Jain DK (2013). In vitro and in vivo evaluation of diclofenac sodium gel prepared with cellulose ether and caropol 934P. Trop J Pharm Res.

[CR8] Chang JY, Oh TK, Choi HG, Kim YB, Kim CK (2002). Rheological evaluation of thermosensitive and mucoadhesive vaginal gels in physiological conditions. Int J Pharm.

[CR9] Chauhan S, Harikumar SL, Kanupriya (2012). Hydrogels: a smart drug delivery system. Int J Res Pharm Chem.

[CR10] Choi HG, Oh YK, Kim CK (1998). In-situ gelling and mucoadhesive liquid suppository containing acetaminophen: enhanced bioavailability. Int J Pharm.

[CR11] Dias RJ, Sakhare SS, Mali KK (2009). Design and development of mucoadhesive acyclovir tablet. IJPR.

[CR12] Dimitrov M, Lambov N, Shenkov S, Dosseva V, Baranovski VY (2003). Hydrogels based on the chemical crosslinked polyacrylic acid: biopharmaceutical characterization. Acta Pharm.

[CR13] El-Leithy ES, Shaker DS, Ghorab MK, Abdel-Rashid RS (2010). Evaluation of mucoadhesive hydrogels loaded with diclofenac sodium–chitosan microspheres for rectal administration. AAPS Pharm Sci Tech.

[CR14] Jadhav UG, Dias RJ, Mali KK, Havaldar VD (2009). Development of in situ-gelling and mucoadhesive liquid suppository of ondansetron. Int J Chem Tech Res.

[CR15] Katzung BG (2004). Basic and clinical pharmacology.

[CR16] Kim C, Lee S, Choi H, Lee M, Gao Z, Kim I (1998). Trials of in situ-gelling and mucoadhesive acetaminophen liquid suppository in human subjects. Int J Pharm.

[CR17] Kuksal A, Tiwary AK, Jain NK, Jain S (2006). Formulation and in vitro, in vivo evaluation of extended-release matrix tablet of zidovudine: influence of combination of hydrophilic and hydrophobic matrix formers. AAPS Pharm Sci Tech.

[CR18] Loya P, Saraf MN (2010). Determination of amtolmetin and its active metabolites in plasma by HPLC-UV: application to a bioequivalence study. J Bioequiv Availab.

[CR19] Matsuda H, Arima H (1999). Cyclodextrins in transdermal and rectal delivery. Adv Drug Deliv Rev.

[CR20] Mekkawy A, Fathy M, El-shanawy S (2013). Formulation and in vitro evaluation of Fluconazol topical gels. Brit J Pharm Res.

[CR29] Ozlem Y, Ilbilge HE, Erde T, Mehmet SD, Ipek T, Arzu B (2013) Tolmetin: an option for multiple NSAID Hypersensitivity in a preschooler. Pediatr allergy Immu pulm 20(3):164–165 10.1089/ped.2012.0185 35923029

[CR21] Park YJ, Yong CS, Kim HM, Rhee JD, Oh YK, Kim CK (2003). Effect of sodium chloride on the release, absorption, and safety of diclofenac sodium delivered by poloxamer gel. Int J Pharm.

[CR22] Pasa G, Mishra US, Tripathy NK, Sahoo SK, Mahapatra AK (2012). Formulation and evaluation of didanosine sustained-release matrix tablets using HPMC K15. Int J Pharm.

[CR23] Prasanna RI, Sankari KU (2012). Design, evaluation and in vitro-in vivo correlation of glibenclamide buccoadhesive films. Int J Pharm Investig.

[CR24] Ramesh N, Socorrina C, Ramakrishna S, Sekar R, Subramania NM (2015). Development of level A in vitro in vivo correlation for Ondansetron hydrochloride sustained release tablet. Indo Am J Pharm Res.

[CR25] Roy S, Pal K, Anis A, Pramanik K, Prabhakar B (2009). Polymers in mucoadhesive drug delivery system. Des Monomers Polym.

[CR26] Shivhare UD, Jain KB, Mathur VB, Bhusari KP, Roy AA, Sharad (2009). Formulation development and evaluation of diclofenacsodium gel using water soluble polyacrylamide polymer. Dig J Nanomater Biostruct.

[CR27] Tasdighi E, Azar ZJ, Mortazavi SA (2012). Development and in-vitro evalution of contraceptive vagino-adhesive propranolol hydrochloride gel. Iran J Pharm Res.

[CR28] Tukker J, Aulton ME (2009). Rectal and vaginal drug delivery. Pharmaceutics, the science of dosage form design.

